# Friction Coefficients for Droplets on Solids: The Liquid–Solid Amontons’ Laws

**DOI:** 10.1021/acs.langmuir.2c00178

**Published:** 2022-03-30

**Authors:** Glen McHale, Nan Gao, Gary G. Wells, Hernán Barrio-Zhang, Rodrigo Ledesma-Aguilar

**Affiliations:** † Institute for Multiscale Thermofluids, School of Engineering, The University of Edinburgh, Edinburgh EH9 3FB, U.K.; ‡ Department of Mechanical Engineering, University of Birmingham, Birmingham B15 2TT, U.K.

## Abstract

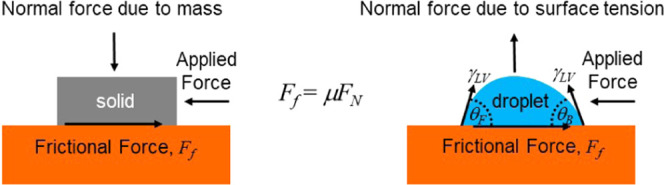

The empirical laws of dry friction between two solid bodies date back to the work of Amontons in 1699 and are pre-dated by the work of Leonardo da Vinci. Fundamental to those laws are the concepts of static and kinetic coefficients of friction relating the pinning and sliding friction forces along a surface to the normal load force. For liquids on solid surfaces, contact lines also experience pinning and the language of friction is used when droplets are in motion. However, it is only recently that the concept of coefficients of friction has been defined in this context and that droplet friction has been discussed as having a static and a kinetic regime. Here, we use surface free energy considerations to show that the frictional force per unit length of a contact line is directly proportional to the normal component of the surface tension force. We define coefficients of friction for both contact lines and droplets and provide a droplet analogy of Amontons’ first and second laws but with the normal load force of a solid replaced by the normal surface tension force of a liquid. In the static regime, the coefficient of static friction, defined by the maximum pinning force of a droplet, is proportional to the contact angle hysteresis, whereas in the kinetic regime, the coefficient of kinetic friction is proportional to the difference in dynamic advancing and receding contact angles. We show the consistency between the droplet form of Amontons’ first and second laws and an equation derived by Furmidge. We use these liquid–solid Amontons’ laws to describe literature data and report friction coefficients for various liquid–solid systems. The conceptual framework reported here should provide insight into the design of superhydrophobic, slippery liquid-infused porous surfaces (SLIPS) and other surfaces designed to control droplet motion.

## Introduction

**Figure 1:**
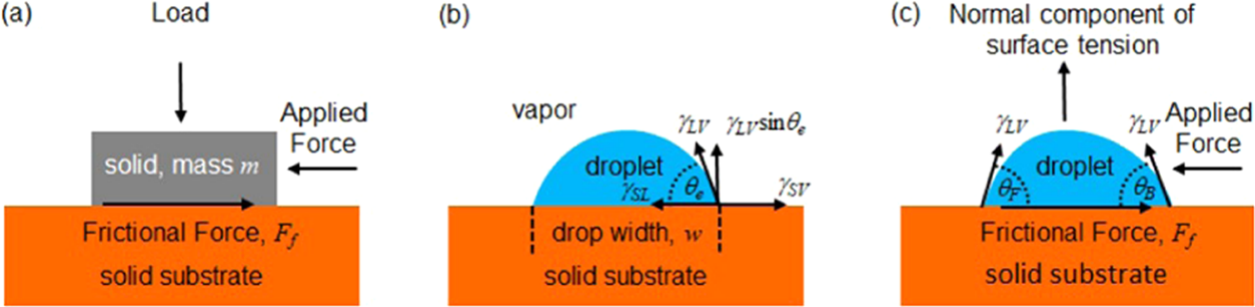
Amontons laws of friction: (a) Solid sliding on a solid due to an applied force. (b) Droplet equilibrium without an applied force. (c) Droplet sliding/rolling on a solid due to an applied force.

Amontons’ first two laws of dry friction state that for any two solid materials, the lateral friction force, *F*_f_, is directly proportional to the normal applied load, *F*_N_, with a constant of proportionality, the friction coefficient μ, that is independent of the contact area (Figure 1a), i.e.
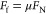
1In the static regime, the maximum frictional force prior to motion is characterized by a coefficient of static friction, μ_s_, that is larger than the coefficient of kinetic friction, μ_k_, in the sliding regime.^1−5^ A third law attributed to Coulomb^6,7^ further states that the coefficient of kinetic friction is independent of sliding velocity, although this is not generally obeyed at higher speeds. While there are limits to the validity of these empirical laws, they, nonetheless, provide a reference point for the dry friction of one solid sliding on a second solid. The language of friction is also very common when dealing with the motion of a droplet on a solid surface, where there is a threshold pinning force and resistance to motion once the droplet is in motion. Overcoming the droplet pinning force has motivated the development of the fields of superhydrophobicity^8^ and, more recently, lubricant-impregnated/liquid-infused surfaces (LIS) and slippery liquid-infused porous surfaces (SLIPS).^9,10^ Understanding low friction droplet motion has been a recent focus in droplet work.^11^ Recently, combined measurements of the resistance force to the movement of a droplet on a range of solid substrates and their geometric shape parameters (front and back contact angles, contact length, contact width) have been reported.^12^ The authors concluded the in-plane frictional force between a liquid drop and a solid can be divided into a static and a kinetic regime in a similar manner to the dry friction of solids. In a separate work, Barrio-Zhang et al.^13^ suggested a direct droplet on solid analogy to eq 1 for the pinning force on a droplet through the use of the normal component of the surface tension force and the contact angle hysteresis. Their approach allows coefficients of static and kinetic friction to be defined for droplets.^11^

In his original work on resistance to droplet motion reported in 1962, Furmidge derived a widely used equation by considering the work done per unit area, γ_LV_(1+cos θ), in advancing a leading edge and dewetting the trailing edge of a droplet.^14^ His work is often reported as
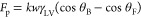
2where *F*_p_ is the lateral (in-plane) force resisting motion, *w* is the droplet contact width, θ_F_ and θ_B_ are the contact angles at the front and rear (back) of the droplet, and *k* = 1 (see also refs^15−18^). As discussed by Krasovitsky and Marmur,^19^ on an inclined plane, θ_F_ and θ_B_ are the contact angles at the stability limits of the respective edges of the droplet, which do not in general simultaneously equal the advancing and receding contact angles, although this is often assumed. In general, *k* is a dimensionless shape factor for the three-phase contact line for which various authors have derived different values, e.g., π/4, 2/π, and 24/π^3^ (see e.g., refs^3, 20^). Presented in this form, the resistive force can be interpreted as the difference in the in-plane components of the surface tension forces at the front and rear of a droplet per unit length multiplied by the droplet perimeter length scaled by a shape factor *k*/π to account for the difference between a two-dimensional (2D) model and a three-dimensional (3D) droplet. Any dependence of eq 2 on the theoretical equilibrium Young’s equation contact angle, θ_e_, given by
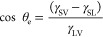
3is implicit. However, since the contact angle given by eq 3 must lie between the receding and advancing contact angles, it seems clear that there should be such a dependence. Recently, we argued that an analogy to coefficients of dry friction for solids can be obtained by a Taylor expansion of eq 2 about an average value, assumed to be θ_e_. To first order, this gives (Figure 1b)^11,13^
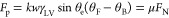
4where the normal component of the interfacial tension force is
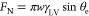
5and the coefficient of droplet friction is defined by
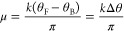
6In this formulation, the relation *F*_p_ = μ*F*_N_ is analogous to Amontons’ first two laws of dry friction for solids, eq 1, but with the normal load force due to gravity replaced by the magnitude of the normal force due to the vertical component of surface tension (Figure 1c). An interesting difference is that for solid friction, the reaction of the surface is to support the load of the solid and is therefore compressive, whereas in the droplet case, the reaction is adhesive and is therefore tensile.

The contact angle hysteresis, Δθ_CAH_ = θ_A_ – θ_R_, determined using volume addition and withdrawal to measure the advancing contact angle, θ_A_, and receding contact angle, θ_R_, gives the maximum range of contact angles in the static regime and, hence, the coefficient of static friction, μ_s_ = *k*Δθ_CAH_/π, at the onset of droplet motion. The difference in dynamic advancing contact angle at the front, θ_A_(*v*), and the dynamic receding contact angle at the rear, θ_R_(*v*), of the droplet, Δθ(*v*) = θ_A_(*v*) – θ_R_(*v*), gives the coefficient of kinetic friction, μ_k_ = *k*Δθ(*v*)/π, for droplet motion and is potentially dependent on the droplet speed, *v*. The advantage of eq 4 to express eq 2 is that it makes explicit the relationship between the in-plane frictional force and both the normal component of the surface tension force and the equilibrium contact angle. It also emphasizes the analogy to Amontons’ first two laws for dry solid friction.

In this work, we show how a surface free energy approach can be used to derive an equation for advancing and receding contact line motion analogous to eq 4 (Amontons-like equation). For droplets, we show this leads to eq 4. This enables coefficients of static and kinetic friction to be defined using the contact angle hysteresis and dynamic contact angles. We show that our Amontons-like equation can accurately describe recent literature data on direct measurements of frictional forces using the complementary measurements of the droplet geometric parameters (front and back contact angles, width, and length) and report the friction coefficients of droplets on surfaces for various liquid–solid systems. We also discuss how Amontons’ second law interpreted as the statement that the coefficients of friction are independent of contact area can be applied to contact line motion, droplet motion, and dry friction for the motion of solids. Finally, we note that a dynamic contact angle in the Amontons-like equation suggests that the frictional force in the kinetic regime is insensitive to droplet speed for low speeds (low capillary number), but at higher speeds (high capillary numbers), it will increase.

## Surface Free Energy and Coefficients of Friction

### Contact Lines

To further understand how an Amontons-like equation can arise for droplets, we consider changes in surface free energy for small advancing or receding displacements of a contact line. These arguments apply to 2D droplets and, because they are local to the contact line, do not depend on the precise profile of the droplet, e.g., whether it is gravitationally flattened or not. A small translation, Δ*r*, of a contact line interchanges solid–vapor, γ_SV_Δ*r*, and solid–liquid, γ_SL_Δ*r*, interfacial energy, and increases (or decreases if Δ*r* < 0) the liquid–vapor interfacial energy by γ_LV_Δ*r* cos θ. The first-order change in the surface free energy, Δ*E*_2D_, as a contact line, is perturbed from its local contact angle, θ, is therefore
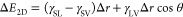
7By requiring this change to vanish, one obtains Young’s law as the equilibrium contact angle, i.e., eq 3.

**Figure 2:**
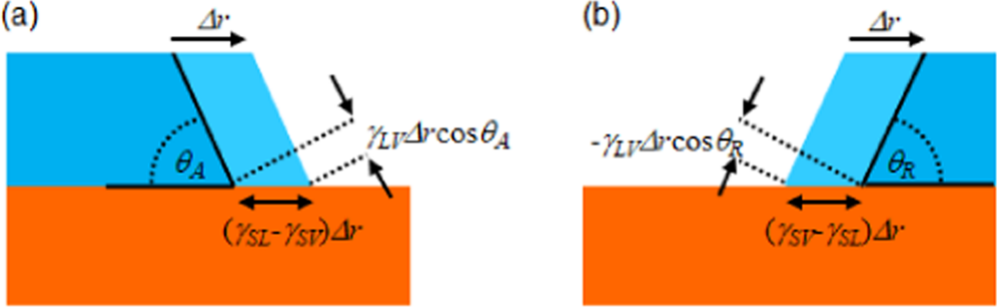
Surface free energy changes: (a) Advancing contact line. (b) Receding contact line.

We now consider an advancing contact line and define an advancing contact angle θ_*A*_ (Figure 2a), with a difference from equilibrium, Δθ_A_ = θ_A_ – θ_e_, so that eq 7 becomes
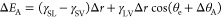
8This can be expanded as
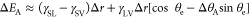
9Using Young’s equation (eq 3) and recognizing that the equilibrium normal component of the liquid–vapor interfacial tension force per unit length of the contact line is *f*_N_ = γ_LV_ sin θ_e_, we find
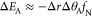
10Similarly, we consider a receding contact angle θ_R_ (Figure 2b) and define the difference, Δθ_R_ = θ_e_ – θ_R_; we obtain
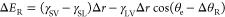
11and so
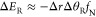
12Because the changes in surface energy given by eqs 10 and 12 are the result of displacements, the corresponding external forces needed to cause such displacements per unit length of the contact line are Δθ_A_*f*_N_ and Δθ_R_*f*_N_. We therefore define coefficients of friction for the advancing and receding contact lines as μ_A_ = Δθ_A_ and μ_R_ = Δθ_R_.

### Droplets

We now consider the advancing and receding contact lines at the front and back of a 2D droplet with contact angles θ_F_ and θ_B_, respectively. In such a case, the leading edge of the droplet advances while the trailing edge recedes. The energy change, Δ*E*_2D_, in translating the position of the droplet by Δ*r* is
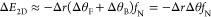
13where Δθ = θ_F_ – θ_B_ is either the contact angle hysteresis for a static droplet or the difference in dynamic contact angles at the front and back edges of a moving droplet.

We now consider a 3D droplet maintaining a circular contact with the solid. Around the front half of the droplet, each point on the contact line advances along the direction of motion (*x*-direction) by the same amount Δ*x*. Similarly, around the back half of the droplet, each point on the contact line recedes along the direction of motion by the same absolute amount. The liquid–solid and solid–vapor area changes in the vicinity of the contact line are given by integrating *r*cosφdφ, where *r* = *w*/2 is the contact radius and φ is the in-plane polar angle around the front (or back) half-perimeter, and this causes a change in the liquid–vapor interfacial energy of 2*r*Δ*x*γ_*LV*_ cos θ_F_ (or 2*r*Δ*x*γ_LV_ cos θ_B_). The total change in surface free energy is
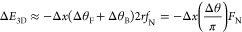
14where *F*_N_ = 2π*r*γ_LV_ sin θ_e_ is the total normal component of the liquid–vapor interfacial tension force around the droplet contact line. Equation 14 suggests the total frictional force is proportional to the total normal force, with a coefficient of proportionality (coefficient of droplet friction) μ = Δθ/π, i.e., *F*_p_ = μ*F*_N_. This is consistent with eq 6 using *k* = 1 derived from Furmidge’s original formulation (eq 2) using a Taylor series expansion.

Generally, we expect the advancing and receding contact angles (or more accurately the cosines) to depend on the position around the droplet perimeter and, once motion begins, the droplet to elongate rather than maintaining a circular contact area, and so introduce an overall constant *k*. The resulting coefficient of static friction for a droplet is therefore
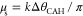
15When a droplet is in motion traveling at a speed *v*, there will be different dynamic contact angles at the front (advancing) contact line θ_A_(*v*) and at the back (receding) contact line, θ_R_(*v*). This leads to the concepts of dynamic advancing and receding coefficients of kinetic friction based upon the dynamic advancing, receding, and equilibrium contact angles. By defining a normal force, *F*_N_ = π*w*γ_LV_ sin θ_e_(*v*), and a difference in dynamic advancing and receding contact angles, Δθ(*v*) = θ_A_(*v*) – θ_R_(*v*), we find a kinetic coefficient of droplet friction, μ_k_
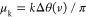
16

## Comparison to Literature Data

To consider the accuracy of our Amontons-like equation for droplets, we can consider whether the geometric parameters measured optically are consistent with direct measurements of in-plane friction forces. A set of data, which is ideal for this purpose, was produced by Gao et al., who reported measurements of the force imparted on a cantilever by a droplet on a moving solid plane.^12^ They used two cameras to simultaneously view the droplet in side profile and parallel to the motion, thereby allowing the geometric parameters of front and back contact angles and droplet contact length and contact width to be measured. Their experiments used droplets of water, hexadecane, and 1-butyl-2,3-dimethylimidazolium bis(trifluoromethanesulfonyl)imide. They used 1*H*,1*H*,2*H*,2*H*-perfluorodecyltrichlorosilane (PFDTS, 96%) to create fluorinated surfaces from silicon wafers, silicone nanofilaments, and SU-8 micropillars (25 μm high, with 50 × 50 μm^2^ top areas and pillar–pillar distance between centers of two adjacent pillars of 100 μm). To create fluorinated TiO_2_ nanoparticle surfaces, they used 1*H*,1*H*,2*H*,2*H*-perfluorooctyltrichlorosilane (PFOTS, 97%). They also used cross-linked PDMS and liquid-like PDMS^21^ surfaces. Full details of their materials and methods are given in the online version of their paper.^12^

**Figure 3:**
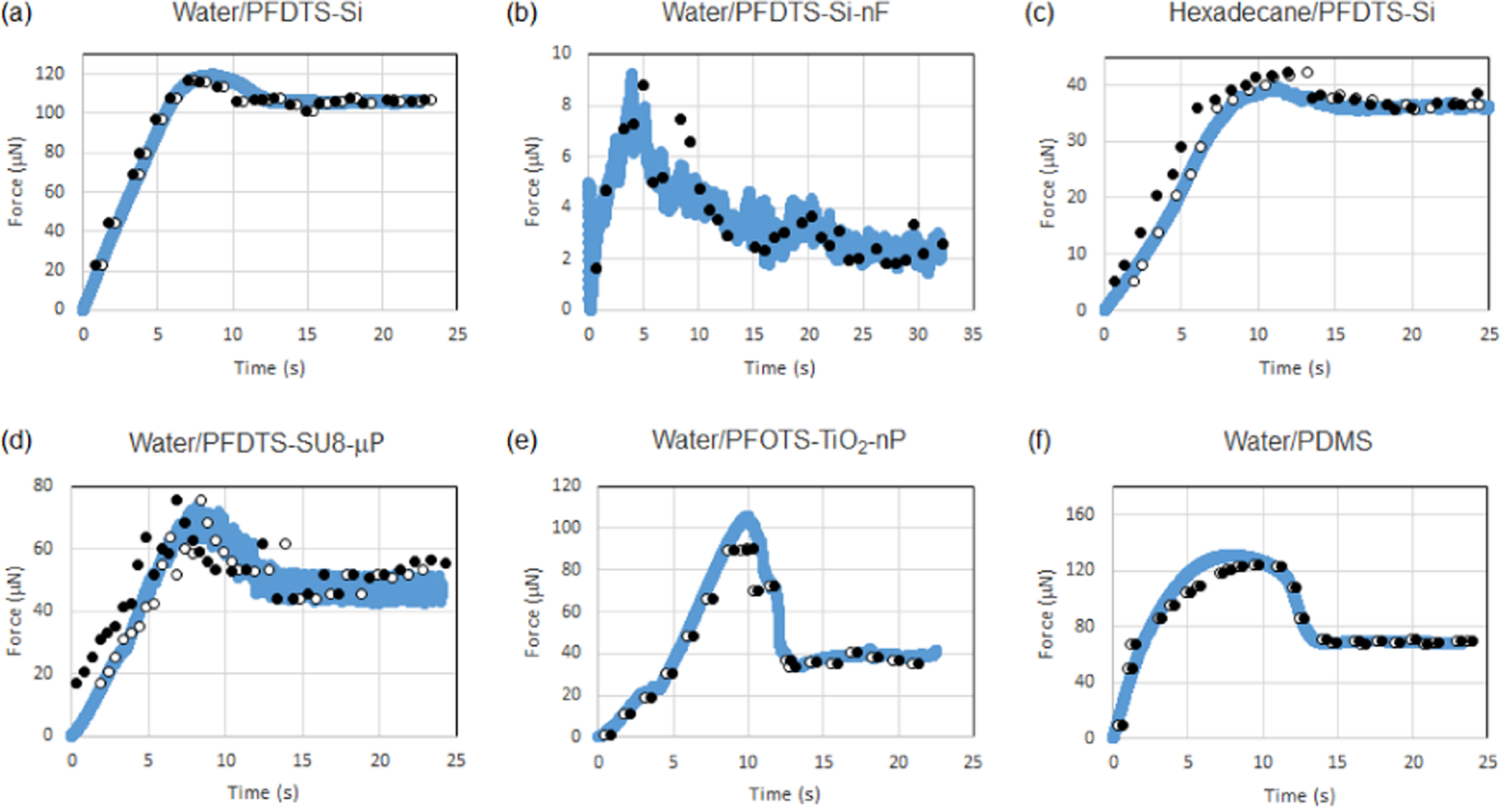
Comparison of the directly measured frictional force (solid line) and the frictional force deduced using the droplet Amontons laws (eq 4) and the measured geometric parameters from droplet images with μ = *k*Δθ/π and *k* = 1.32 for data in panels (a)–(e) and *k* = 1.04 for data in panel (f). The solid symbols (•••) are without offsets to the time axes, and the open symbols (○○○) use offsets to the time axes of 0.45, 0.00, 1.20, 1.50, −0.4, and −0.3 for data in panels (a)–(f), respectively.

We start by analyzing the data from their supplementary information for droplets of water on fluorinated silicon (Water/PFDTS-Si), superhydrophobic fluorinated silicone nanofilaments (Water/PFDTS-Si-nF), fluorinated SU-8 micropillars (Water/PFDTS-SU8-μP), fluorinated TiO_2_ nanoparticles (Water/PFOTS-TiO_2_-nP), and PDMS (Water/PDMS), and for droplets of hexadecane on fluorinated silicone (hexadecane/PFDTS-Si) (i.e., Figures S5, S6, S8–S10, and S7 in their Supporting Information). In each case, the substrates were translated at a constant speed of approximately 200 μm/s. The surface tension of water and hexadecane are γ_LV_ = 72.8 and 27.5 mN/m, respectively. The droplets initially have similar contact length, *l*, and contact width, *w*, but as a droplet is forced into motion, its length can become up to 20% larger than its width. In our theory, we have generally assumed a circular contact area, whereas droplets in motion can have a significantly elongated droplet–solid contact shape. Here, we assume the contact width and length define an ellipse and use Ramanujan’s formula^22,23^ to calculate an approximate equivalent circular contact diameter, *w*_equiv_, with the same perimeter length
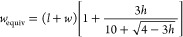
17where *h* = (*l* – *w*)^2^/(*l* + *w*)^2^. We then use an average of the front and rear forces to estimate the average normal component of surface tension force per unit length along the droplet perimeter
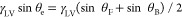
18Figure 3 shows the experimentally measured frictional force data as a solid line. The optically measured contact angles at the front and rear of the droplet and the droplet contact length and width data used in eq 4 are shown by the solid symbols (•••). For these data points, a value of *k* = 1.32 has been used in all data sets apart from Water/PDMS, where a value of *k* = 1.04 has been used to match to the measured frictional force during steady droplet motion toward the end of each time sequence. In all six cases, eq 4 captures the shape of the frictional force with time, but in three cases, the calculated force systematically overestimates the measured value in the static regime below the peak in the force. The most obvious example is for Water/PFDTS-SU8-μP, where at time *t* = 0, the calculated force is clearly offset from the measured force (Figure 3d).

To match up each time sequence, we therefore assume a small time offset between the frictional force and the estimates from eq 4 using the optical geometric measurements. These offset time data sequences are shown by the open symbols (○○○) in Figure 3 and correspond to offsets of between −0.4 and 1.5 s (Note that for Water/PFDTS-Si-nF, the solid symbols overlay and obscure the open symbols because no offset was required). In the case of Water/PDMS where a lower value of *k* = 1.04 was required to match the kinetic regime, the peak in the force curve separating the static and kinetic regimes is more rounded and extended than in the other data sets. It appears likely both this feature and the lower value of *k* are because of the softness of the PDMS substrate compared to the other substrates, which are rigid. We conclude from these data sets that the droplet form of Amontons’ laws (eq 4) is in excellent agreement with the experimental data, provided one assumes a small offset in the time axes.

We now discuss the possible causes of an offset in the time axes, which is required primarily to match the data in the early time static regime period in Figure 3c,d. The experimental method used two cameras to measure geometric parameters and also recorded the frictional force *via* the deflection of a hollow rectangle glass capillary inserted into the center of the droplet. The matching of the three time series used the end of data capture for each run, and so there is a possibility of a slight mismatch. It is also possible that the distortion of the droplet shape or the methods to estimate contact angles in the static and dynamic regimes might cause offsets. In addition, when the stage is in motion, the position of the capillary within the droplet moves during an initial period to the front edge of the droplet (the only exception is for the superhydrophobic case of Water/PFDTS-Si-nF); the change in the relative position of the capillary in the droplet and the linked deformation of the liquid–vapor interface is visible in the supplementary videos provided with the published paper reporting the original data. The speed of translation of the substrates and the droplet spherical radius provide timescales larger than that needed for the offset in time axes in the fitting in Figure 3. This rearrangement of the relative position of the capillary when measuring force is a complication not present in the analogous experiments of friction with a sliding solid. While we cannot be certain about the cause of an offset in the time axes for the droplet experiments, it is plausible that offsets may be subtly within the experimental method.

**Figure 4:**
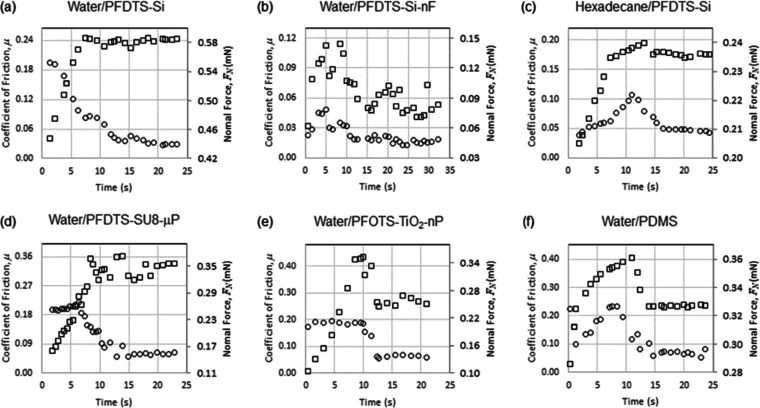
The coefficients of friction (□□□□ symbols—left-hand axes) and the normal component of the surface tension force (○○○ symbols—right-hand axes) for the data in Figure 3.

In Figure 4, we show the time sequences for the coefficients of friction, μ = *k*Δθ/π, and the normal component of surface tension force, *F*_N_ = π*w*_equiv_γ_LV_ sin θ_e_, corresponding to the data in Figure 3. From these plots, we identify the maximum value during the initial increase in μ as the coefficient of static friction, μ_s_, and the average value during steady-state motion after the peak as the coefficient of kinetic friction, μ_k_, (Table 1). Due to the time resolution in the measurement of the geometric parameters, the data cannot capture narrow peaks, and so we cannot provide an uncertainty estimate beyond noting the coefficient of static friction is likely to be an underestimate. In contrast, the coefficient of kinetic friction can be taken as an average over a period of time when it is approximately constant, and this allows an estimate of its uncertainty. To place the magnitude of these coefficients of friction into the context of solids, the coefficient of friction for Teflon sliding on Teflon^24,25^ is 0.05 and for aluminum magnesium chloride (AlMgB_14_) (also known as BAM), reported to be the world’s slipperiest solid material, is 0.04–0.05 (unlubricated) in tests in diamond tip nano-scratch tests.^26^

**Table 1:** Coefficients of Static and Kinetic Friction for Various Droplet/Solid Systems

system	μ_s_	μ_k_
water/PFDTS-Si	0.244	0.240 ± 0.004
water/PFDTS-Si-nF	0.114	0.056 ± 0.014
hexadecane/PFDTS-Si	0.196	0.176 ± 0.006
water/PFDTS-SU8-μP	0.362	0.340 ± 0.040
water/PFOTS-TiO_2_-nP	0.428	0.266 ± 0.016
water/PDMS	0.404	0.236 ± 0.002

**Figure 5:**
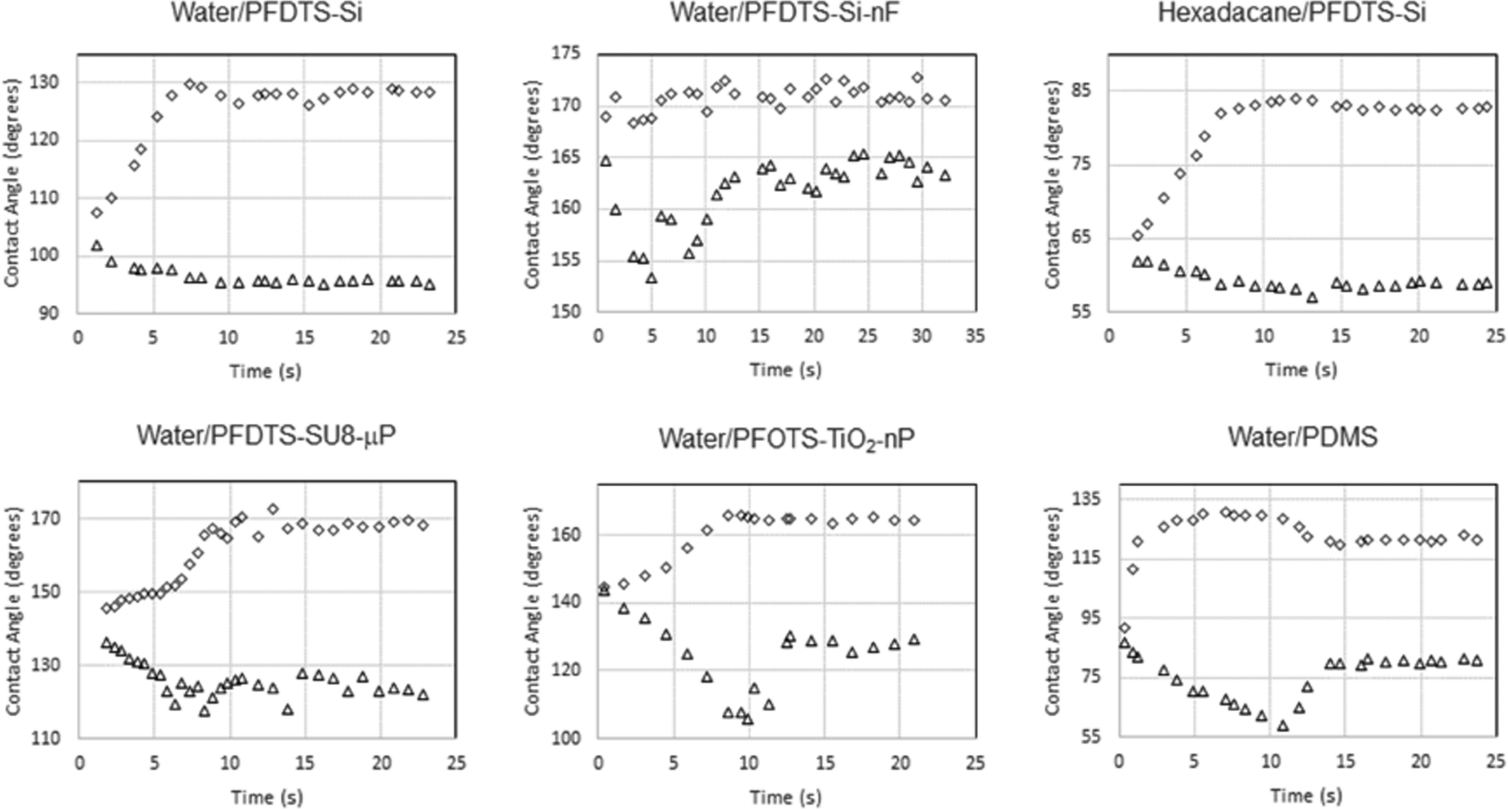
The front contact angle (◊◊◊ symbols) and rear contact angle (ΔΔΔ symbols) for the data in Figure 3.

In Figure 5, we reproduce the time sequences for the front and rear (back) contact angles corresponding to the data in Figure 3. Apart from water on PDMS, the front contact angle always increases to a maximum and then stabilizes rather than decreasing in the kinetic regime (to within the measurement accuracy). In contrast, the rear contact angle decreases and either stabilizes or increases before stabilizing in the kinetic regime. The difference between the static and kinetic coefficient of friction, where there is one, is therefore determined by the contact line motion at the back of the droplet. To quote Extrand and Gent, “*Simultaneously, the front edge of the drop tends to creep forward slightly, increasing the length of the drop. But the entire drop does not move until a critical force F is applied*.”^20^ The difference in motion of the advancing and receding contact lines of a droplet on surfaces has been discussed by a range of authors (e.g., refs^19, 21, 27−30^). For example, one difference for the receding contact line on a micropillar superhydrophobic surface compared to the advancing contact line is that it can dewet a micropillar by the formation and rupture of a capillary bridge,^31^ which is also a mechanism that is applicable to other types of pinning features on surfaces. It is further known that droplets break at the rear contact line upon advancing when the receding contact angle approaches zero.^20^

**Figure 6:**
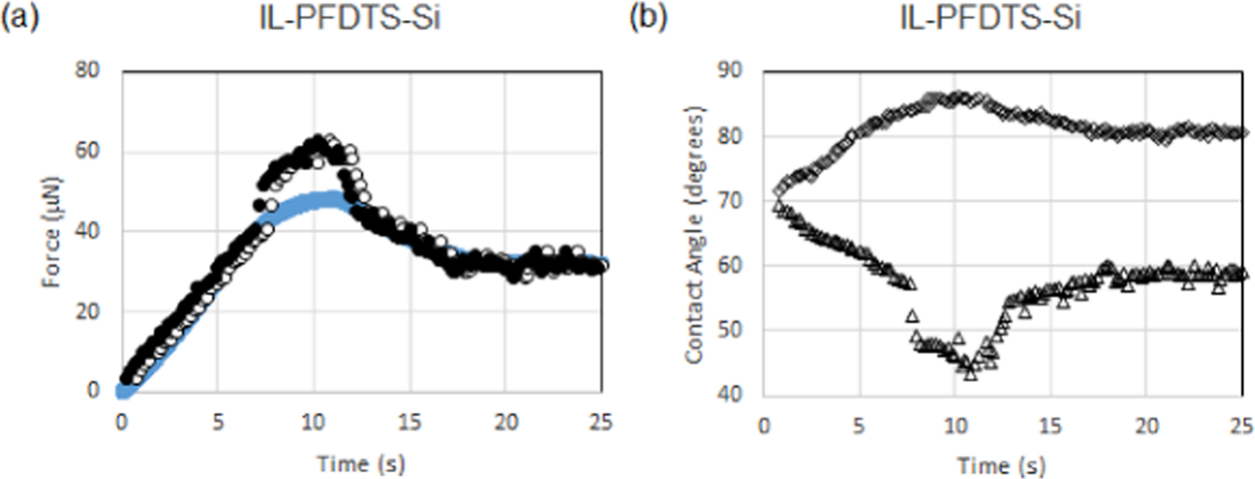
Ionic liquid on fluorinated silicon (IL-PFDTS-Si): (a) Comparison of the measured frictional force (solid line) to the force deduced from geometric parameters with *k* = 1.13 without offsets (••• symbols) and with an offset to the time axes of 0.6 s (○○○ symbols). (b) Front contact angle (◊◊◊ symbols) and rear contact angle (ΔΔΔ symbols).

Gao et al.^12^ also considered the frictional forces on an ionic liquid (1-butyl-2,3-dimethylimidazolium bis(trifluoromethanesulfonyl)imide) with a surface tension of γ_LV_ = 34.6m N/m on a fluorinated silicon (IL-PFDTS-Si) substrate translated at a constant linear velocity of approximately 200 μm/s. To fit the data for the kinetic regime requires *k* = 1.13 (solid symbols ••• in Figure 6a) with an offset to the time axes of 0.6 to bring the majority of the static regime data into an agreement (open symbols ○○○ in Figure 6a). Fitting this data assuming *k* = 1.32 to be consistent with the five data sets in Figure 3 for droplets on rigid substrates overestimates the force in the kinetic regime and would require an offset of ca. 5 μN to overlay the majority of the data, with the exception of the data in the peak region. The data for the front and rear (back) contact angles of the droplet shows that the behavior of the rear contact angle differs from other data sets in this peak region (Figure 6b), with a step decrease of around 10° occurring after 7 s and before the peak force at c.a. 10 s. This suggests that this experiment might have been influenced by some pinning defects on this particular sample.

Gao et al.^12^ measured the friction force as a function of time for a ca. 1.0 μL droplet of water on liquid-like PDMS, where, unlike other surfaces, the maximum frictional force and the frictional force during steady motion (251 μm/s) were found to be equal. However, their data does not include any of the droplet geometric measurements (front and back contact angles, contact width, and contact length). These “liquid-like” PDMS samples were prepared following the method reported by Krumpfer and McCarthy.^21^ In these surfaces, only one end of the PDMS is covalently grafted on the substrate, with the remaining part of the PDMS keeping its high mobility with rotational and/or bending motion. Using the information in Pilat et al.*,*^32^ we can estimate the advancing and receding contact angles for water on these surfaces θ_A_ = 105 ± 2° and θ_R_ = 93 ± 3° using the volume addition and withdrawal method and so the contact angle hysteresis is Δθ_CAH_ = 12 ± 5°. By assuming that the droplet is approximately a spherical cap shape, we can estimate the contact width from the droplet volume and an average equilibrium contact angle. This allows us to estimate the frictional force from eq 4 using *k* = 1.32 is (14 ± 6) μN, consistent to within error of the measured friction force of 15.1 μN in the kinetic regime. This also implies a static coefficient of friction for this liquid-like surface of μ_s_ = 0.09 ± 0.04. A single universal value of *k* = 1.32 therefore appears to be consistent with the data in Figure 3 from Gao et al.^12^ for the five rigid substrates, provided one allows for an offset in the time axes.

Wang and McCarthy^33^ also reported an alternative slippery omniphobic covalently attached liquid (SOCAL) surface obtained through acid-catalyzed graft polycondensation of dimethyldimethoxysilane with extremely low CAH (≤1°) for liquids that span surface tensions from 78.2 to 18.4 m/Nm (for a discussion of the liquid-like nature of surface-tethered PDMS brushes see ref^34^). Using *k* = 1.32, their paper implies coefficients of static friction of μ_s_ = 0.007, 0.007, 0.001, 0.003, 0.001, <0.001, and <0.001 for water, diiodomethane, toluene, hexadecane, cyclohexane, decane, and hexane, respectively. Measurements reported by Barrio-Zhang et al.^33^ for water on SOCAL report a contact angle hysteresis of Δθ_CAH_ = 2.5 ± 1.7°, giving an estimated coefficient of static friction of μ_s_ = 0.018 ± 0.012. Such low coefficients are comparable or lower than the coefficients of static friction reported for the most slippery solid-on-solid systems (i.e., Teflon and BAM).

## Discussion of Amontons’ Laws in a Droplet Context

Gao et al.^2^ provide a historical review of Amontons’ laws from studies of the force required to slide a solid object on a solid surface, starting with the conclusions of Leonardo da Vinci that the friction force doubled when the weight (normal externally applied load, *F*_N_) was doubled and, second, that the (lateral) friction force, *F*, was independent of the way the objects were positioned on the surface (i.e., that the force did not depend on the area of contact, *A*, between the moving surfaces). They note that these observations were later confirmed by Amontons (1663–1706), and that Coulomb further noted the velocity independence of the friction force. Mathematically, this is summarized as a friction coefficient, μ = *F*/*F*_N_, which is independent of the “apparent” or macroscopic contact area and sliding velocity of the load.

A striking visual demonstration of Amontons’ second law is to use a solid block with one face much smaller than the other and show that the friction force is independent of whether it is placed on its largest or smallest surface area face. In the droplet case, because the material is a liquid, one cannot change the contact area between the droplet and the solid in such a manner. To change the area, one would need to change the droplet volume or the equilibrium contact angle (through either the surface chemistry or roughness/topography). As stated above, da Vinci’s second observation cannot be applied to droplet friction. However, it remains the case that one can state that for a droplet on a solid, the (lateral) friction force is directly proportional to the normal component of surface tension force (first law) with a constant of proportionality, the coefficient of friction, that is constant and independent of the contact area (second law). A key part of this statement is that it is the coefficient of friction, μ, which is independent of the contact area, and this does not include statements about the orientation of a rigid object.

In considering the droplet form of Amontons’ third/Coulombs law, eq 4 provides insight beyond eq 2 derived by Furmidge. Specifically, it provides an explicit dependence on the contact angle through the normal component of the surface tension force and is in a separable form with a contact angle hysteresis factor. From the perspective of designing a superhydrophobic surface, eq 4 encapsulates the idea that a surface with a high equilibrium angle will give a low normal component of the surface tension force, but the contact angle hysteresis will determine the coefficient of static friction and whether it is a so-called “sticky” or “slippery” superhydrophobic surface. From the perspective of designing lubricant-impregnated or slippery liquid-infused porous surfaces, eq 4 encapsulates the idea that a sufficiently low contact angle hysteresis will give a low coefficient of static friction. On these surfaces, drop motion can be easily initiated without the need for high equilibrium contact angles to achieve a low normal component of the surface tension force. The recognition that the coefficient of kinetic friction can be different from the coefficient of static friction is a reminder that designing a surface on which droplet motion can be easily initiated may not be the same as designing a surface that has dynamic drop mobility. This appears relevant to liquid-like surfaces, such as SOCAL.

One can also hypothesize that the normal component of surface tension force should use the dynamic contact angle, θ_D_(Ca), which in the Cox–Voinov theory^35,36^ is predicted as a function of the speed of the contact line, *U*, by
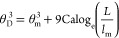
19where Ca = η*U*/γ_LV_ is the capillary number, and η is the viscosity of the droplet. For an advancing contact line, we assume that the microscopic contact angle is θ_m_ = θ_A_. For a receding contact line, eq 19 is also valid,^36^ where we assume θ_m_ = θ_R_ and let Ca → −Ca. In eq 19, the logarithmic term uses a microscopic length *l*_m_ and a typical macroscopic length scale at which the dynamic contact angle is measured.^37^ For small droplets, *L* is often taken as the capillary length *l*_c_ = (γ_LV_/ρ*g*)^1/2^, where ρ is the density of the droplet and *g* = 9.81 m/s^2^ is the acceleration due to gravity. eq 19 suggests a low-speed regime where the dynamic contact angle remains approximately constant, i.e., when |Ca| ≪ θ_m_^3^/9 log_e_(*L*/*l*_m_). Beyond this limit, the dynamic contribution in eq 19 can be used to estimate the kinetic coefficient of friction. Using a Taylor expansion around Ca = 0, we expect the frictional coefficient arising from Cox–Voinov theory to vary linearly with the interface speed to first order, i.e.
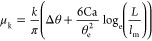
20We hope that such considerations on the possible velocity dependence of the coefficient of kinetic friction will provide motivation for future experiments to simultaneously measure the friction force and the geometric parameters of the droplet, particularly in the kinetic regime.

Beyond droplets, it is possible that our ideas on the coefficient of friction for advancing and receding contact lines will be relevant to the consideration of liquid friction on the microscale, for example, in the context of the molecular kinetic theory^38,39^ and to molecular dynamics simulations of wetting.^40,41^ We believe there will also be broader relevance to macroscopic processes and other systems, e.g., porous media and capillary imbibition.^42^ Our work does not address microscopic models, such as the Prandlt–Tomlinson model,^43−46^ which might provide a complementary approach to friction on rough/textured and chemically heterogeneous surfaces where capillary bridges may form and break as the droplet dewets successive features.^31,47,48^

## Conclusions

In this work, we have developed the concept of coefficients of static and kinetic friction for contact lines and droplets. We have shown that a surface free energy approach can produce an equation and laws analogous to Amontons’ first and second laws of dry solid friction with the in-plane frictional force proportional to the normal component of the surface tension force and a constant of proportionality *k*Δθ/π. We have shown these laws are consistent with eq 2 relating advancing and receding contact angles to the pinning force on a droplet. We have compared the prediction of these new liquid–solid Amontons-like laws against recent experimental measurements, reporting for the first time the friction coefficients of droplets on surfaces for various liquid–solid systems. We have also suggested that Amontons’ third law/Coulombs law may be considered within a model of coefficient of kinetic friction and dynamic contact angles. Our work provides a conceptual framework linking droplet and contact line friction to solid-on-solid friction and provides a unified approach to considering Furmidge’s equation for droplet pinning and droplet friction.
